# Control Parameters Optimization Based on Co-Simulation of a Mechatronic System for an UA-Based Two-Axis Inertially Stabilized Platform

**DOI:** 10.3390/s150820169

**Published:** 2015-08-14

**Authors:** Xiangyang Zhou, Beilei Zhao, Guohao Gong

**Affiliations:** 1 School of Instrumentation Science and Opto-electronics Engineering, Beihang University (BUAA), Beijing 100191, China; E-Mail: zhaobeilei1992@buaa.edu.cn; 2 Beijing Aerospace Era Laser Navigation Technology Co., Ltd., Beijing 100094, China; E-Mail: gongguohao_2008@yeah.net

**Keywords:** unmanned airship, two-axis inertially stabilized platform, mechatronic system co-simulation, virtual prototyping, control parameters

## Abstract

This paper presents a method based on co-simulation of a mechatronic system to optimize the control parameters of a two-axis inertially stabilized platform system (ISP) applied in an unmanned airship (UA), by which high control performance and reliability of the ISP system are achieved. First, a three-dimensional structural model of the ISP is built by using the three-dimensional parametric CAD software SOLIDWORKS^®^; then, to analyze the system’s kinematic and dynamic characteristics under operating conditions, dynamics modeling is conducted by using the multi-body dynamics software ADAMS™, thus the main dynamic parameters such as displacement, velocity, acceleration and reaction curve are obtained, respectively, through simulation analysis. Then, those dynamic parameters were input into the established MATLAB^®^ SIMULINK^®^ controller to simulate and test the performance of the control system. By these means, the ISP control parameters are optimized. To verify the methods, experiments were carried out by applying the optimized parameters to the control system of a two-axis ISP. The results show that the co-simulation by using virtual prototyping (VP) is effective to obtain optimized ISP control parameters, eventually leading to high ISP control performance.

## 1. Introduction

An inertially stabilized platform (ISP), which is used to support and stabilize the imaging loads so that the sensor’s line of sight (LOS) can track the target accurately in real-time, plays an important role in aerial remote sensing. In recent years, due to their potential applications in different fields, great interest in the utilization of unmanned aerial vehicles (UAVs) has arisen [1,2,3,4,5,6]. The reason why unmanned airships (UAs) have become the ideal platform is mainly because they are less intrusive, noiseless, capable of hovering, less energy consuming, cost efficient and able to stay in the air for longer periods [2,6]. Although robotic airships have some advantages over other UAVs at low speeds and low altitude applications, they present a challenging control problem too. Furthermore, their kinematic and dynamic models are highly nonlinear and coupled [4]. Similarly, there are some disadvantages in stabilization when UA is used as aviation platform in an aerial remote sensing system, such as easily pitch maneuvering, low leveling stabilization, heading sideways swinging and so on. Therefore, higher requirements are put forward for the control system when an ISP is supported by an UA platform.

In order to achieve the tracking accuracy and stabilization required by an UA-based aerial remote sensing system, the ISP control system needs to have more accurate and reliable system parameters under the condition of supporting large loads and being installed on an unsteady suspension pod. Generally, to optimize the control system parameters, many complicated, repeated and rigorous experiments have to be carried out on the ground by simulating the aircraft environment. Moreover, in order to ensure the robust adaptability of the control system in a real environment, the electrical and mechanical cooperation debugging process is crucial. In the conventional design process, the structural system modeling is usually simplified, which easily decreases the fidelity of the simulation. In addition, since much time is required by the parameter optimization and reliability testing process of the control system, this can potentially damage the ISP. As a result, it is hard to obtain good control performance when traditional methods are employed. Therefore, to improve the performance of an ISP’s control system, control parameter optimization based on co-simulation using virtual prototyping is indispensable.

In order to significantly reduce the amount of physical testing that is required, virtual prototyping (VP) can be used, especially in the design stage [7]. VP is a process used for shortening the time to market and reducing the product cost. It makes use of a digital model for testing and evaluating the specific characteristics of a product and for simulating the manufacturing processes in a computational environment [8]. Physical prototyping, although is more desirable, may prove to be expensive and time consuming [7]. Repeated, efficient, and extensive use of prototypes is a vital activity that can make the difference between successful and unsuccessful entry of new products into the competitive world market [9]. The value of VP is rapidly being recognized for a wide range of engineering applications. These applications range from illustrating the potential of a system early in the technology assessment activity or early developmental phase to detailed analysis of mature designs in the advanced engineering phase [10]. ADAMS™ is a successful software for VP analysis [11].

The co-simulation technique based on ADAMS™ and SIMULINK^®^ cooperation can be useful tool for improving the development cycle, which is suitable for the design of a mechatronic system with a complex mechanical structure and dynamic behavior with a control system [12,13]. MATLAB^®^ and SIMULINK^®^ focus on different areas and, if used in isolation, are unable to provide satisfactory answers to all the questions involved in the global simulation of mechatronic systems [14]. The control module is generated in ADAMS™, which is the interface between ADAMS™ and MATLAB^®^, and contacts the model in ADAMS™ with control system in MATLAB^®^ [11,15]. The method of co-simulation using ADAMS™ and MATLAB^®^ provides a new method for studying the dynamics of complex systems, which can simplify the simulation process and make the simulation results more accurate and increase the design reliability [16]. Co-simulation is not only widely used for verification of the control strategy of complicated dynamic systems, but also used for controller designing for dynamic systems [17]. In [18], it was shown that the use of co-simulation for robot design was more efficient than without co-simulation. The efficiency of co-simulation techniques for the development of mechatronic systems has been proven use [12]. Interactive co-simulation of ADAMS™ and MATLAB^®^ could help designers consider the two prime parts of mechanics and control, and consequentially result in higher design efficiency [19].

In this paper, to meet the high precision and high stability requirements of an UA-based two-axis ISP for remote sensing, control parameter optimization based on co-simulation of the mechatronic system is carried out using ADAMS™ and SIMULINK^®^ cooperation. The purpose is to improve the control performance of the ISP by utilizing the significant characteristics of co-simulation and virtual prototyping. The dynamics prototype is established in the ADAMS™ environment instead of using the simplified model of traditional design, whose dynamic parameters are then used as inputs for a SIMULINK^®^ controller to optimize the control system parameters. As a result, a control system with high precision and high stability characteristics can be achieved. To verify the methods, experiments are carried out by applying the optimized parameters to the real control system of a two-axis UA-based ISP.

## 2. Background Analysis

### 2.1. Aerial Remote Sensing System

**Figure 1:**
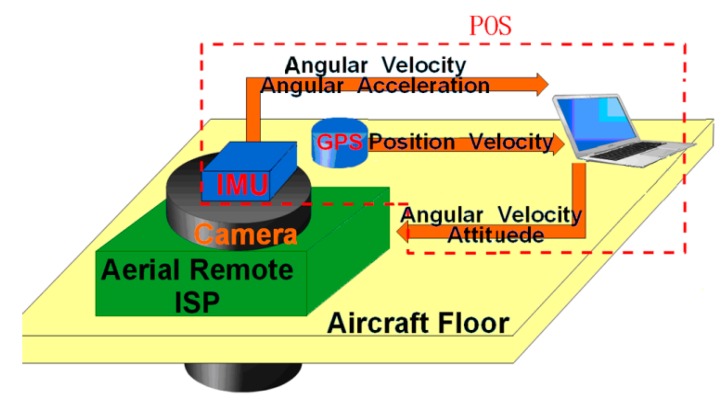
Schematic diagram of an aerial remote sensing system.

Figure 1 shows the schematic diagram of an aerial remote sensing system. Generally, an aerial remote sensing system consists of four main components [20,21], *i.e.*, a multi-axis ISP, an imaging sensor, a position and orientation system (POS) and the aircraft vehicle. When applied, the multi-axis ISP is mounted on the aviation platform, and the imaging sensor and POS are fixed on the inner of the ISP’s gimbals. When the aviation platform rotates or jitters, the control system of multi-axis ISP gets the high-precision attitude reference information measured by the POS and then routinely controls the LOS of the imaging sensor to achieve accurate pointing and stabilization relative to the ground level and flight track.

### 2.2. Working Principle of Two-Axis ISP

**Figure 2:**
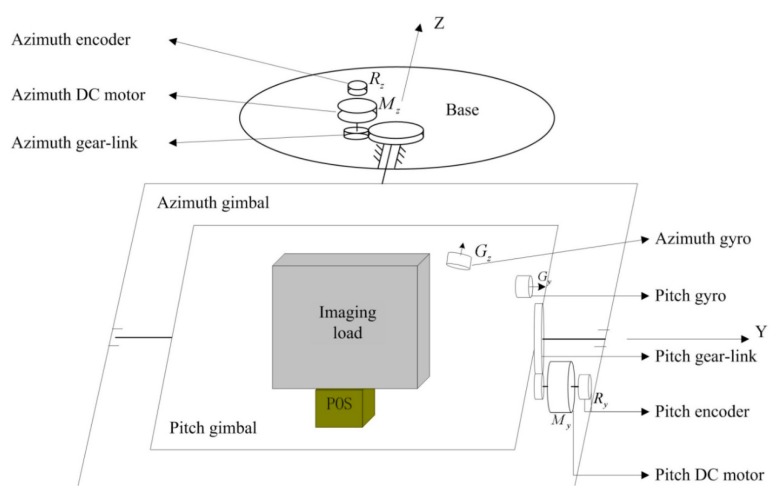
Schematic diagram of a two-axis ISP’s working principle.

Figure 2 shows the schematic diagram of the two-axis ISP’s working principle. We can see that the ISP consists of two gimbals, which are an azimuth gimbal (A-gimbal) and a pitch gimbal (P-gimbal) [20]. The P-gimbal is assembled on the A-gimbal and can rotate around the Y_p_ axis. The A-gimbal is assembled on the base of the aviation platform and can rotate around the Z_a_ axis. G_p_ and G_a_ stand for the rate gyros that measure the inertial angular rate of the P-gimbal and A-gimbal, respectively. E_p_ and E_a_ respectively stand for the photoelectric encoders installed on the P-gimbal and A-gimbal, which are used for measuring the relative angles between gimbals. M_z_ and M_y_ respectively stand for the gimbal servo motors which drive the A-gimbal and P-gimbal to keep them steady in inertial space. Ay represents the accelerometer installed on the P-gimbal that is used to measure the gimbal’s rotary angular acceleration.

### 2.3. Three Closed-Loop Compound Control Scheme

**Figure 3:**
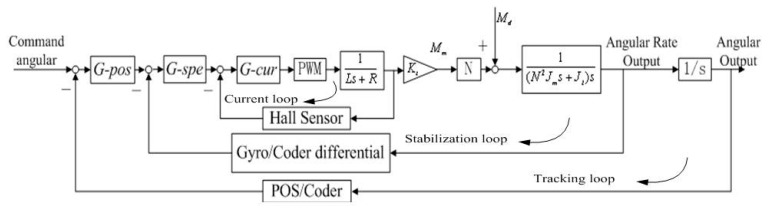
A block diagram of a traditional three-loop control system for an ISP.

Figure 3 shows the block diagram of a three-loop control system for an ISP. Conventional stabilization techniques employ rate gyros, rate integrating gyros, or rate sensors to detect angular rate disturbances of the LOS [22]. The blocks of G-pos, G-spe and G-cur separately represent the controllers in the position loop, speed loop and current loop; the PWM block represents the power amplification used for amplifying the current to drive the torque motor; L represents the inductance of a torque motor and R represents the resistance; K_t_ represents the torque coefficient of the motor and N is the transition ratio from the torque motor to the gimbals; J_m_ represents the moment of inertia of the motor and J_l_ represents the moment of inertia of the gimbals along the rotation axis.

Since the maximum required velocity of an ISP is usually low, and rarely exceeds 100 degrees per second, gearing can be considered in an attempt to reduce the size and weight of the actuator, particularly when the torque requirements are demanding [23]. Thus, to meet the requirements of high driving torque, heavy load and small size, *etc.*, the gimbals are often designed to be rotated by indirect drive motors linked to each gimbal through gear trains. To satisfy the high quality imaging requirements, the ISP needs to realize high pointing precision without jitter under lower tracking speed. However, since most gearing arrangements inevitably introduce additional friction and torsional resonances in the system, the reaction torque from a geared actuator constitutes an equivalent torque disturbance that can degrade the stabilization performance [23].

Although requirements for ISPs vary widely depending on the application, they all have a common goal, which is to hold or control the line of sight (LOS) of one object relative to another object or inertial space [23]. It should be noted that in this paper, the design of an ISP system is subjected to experimental requirements in both pointing and stabilization accuracy of the whole remote sensing system. To meet the requirements of high driving torque capacity, gear trains are employed in a two gimbals drive system. Therefore, for the designed system, it is difficult to obtain a higher accuracy level compared to a direct drive system since there will be errors derived from the gear train.

## 3. Three-Dimensional CAD Modeling

### 3.1. The Structure of ISP System

**Figure 4:**
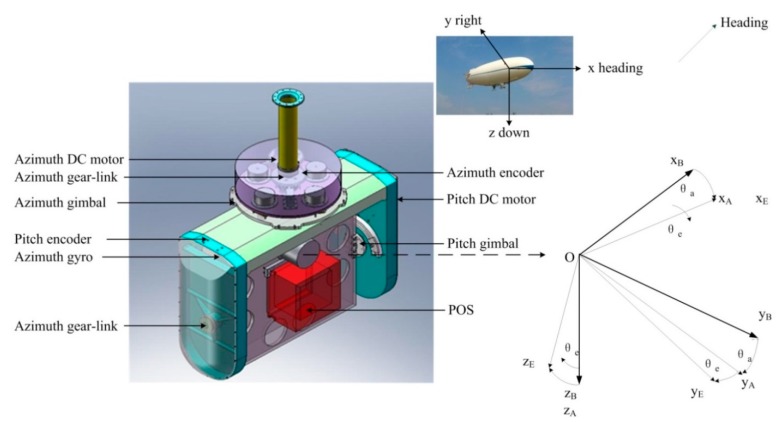
Three-dimensional CAD structural model of a two-axis ISP built using SOLIDWORKS^®^.

Figure 4 shows a three-dimensional CAD structural model of a two-axis ISP built using the three-dimensional parametric CAD software SOLIDWORKS^®^. The model represents a compromise between a series of conflicting requirements, such as small size and heavy load capacity, light weight and high stiffness, *etc.*, which has to undergo appropriate modifications to realize optimization by adjusting parameters. It can been seen that the two-axis ISP is mainly made up of five sub-systems, *i.e.*, shaft supporting load system, gimbal structure system, control system, inertial measurement system and drive-transmission system, by which the main function of disturbance rejection can be realized in real-time. As a result, the LOS are kept tracking the target in the directions of both the azimuth and pitch axes all along.

### 3.2. FEM Analysis

#### 3.2.1. Static Analysis Results

**Figure 5:**
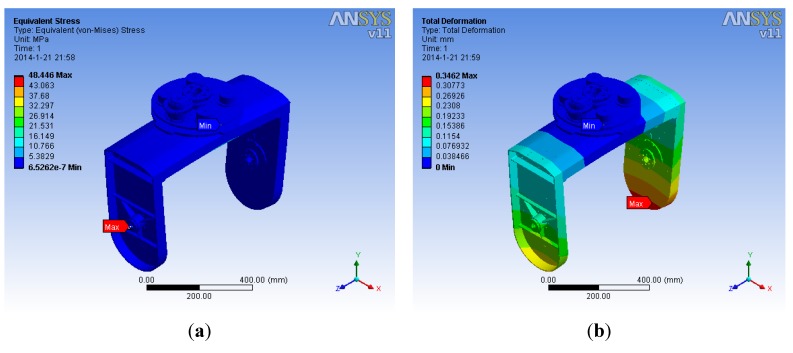
FEA static results: (**a**) Displacement distribution and (**b**) Stress distribution.

To validate the static and dynamic performance, finite element analysis (FEA) is performed by the ANSYS Workbench Platform (AWP). The FEA model was directly imported from the established SOLIDWORKS^®^ model which is then meshed by using the highly-automated AWP meshing function. Figure 5 shows the FEA static results that satisfy the strength design requirements.

#### 3.2.2. Dynamic Analysis Results

Modal analysis can provide a bidirectional connection with all major CAD systems, enabling more efficient simulation-driven design decisions. A structural mode can be thought of as a shape and a frequency at which a structural shape resonates [23]. Modal analysis has become a major technology in the quest for determining, improving and optimizing the dynamic characteristics of engineering structures [24]. The fundamental principles of modal FEM analysis can be simply generalized as follows [25,26]:

The modal representation of a mechanical structure can be determined analytically if a lumped mass-spring system is concerned. In the general case of a continuous structure, a numerical approximation by means of a Finite Element Model (FEM) is made, discretizing the structure in a finite number of physical coordinates. The equations of motion describing this approximated system in the time and Laplace domain are given by:

(1) $$\left[M]\{\ddot{x}(t)\}+[C]\{\dot{x}(t)\}+[K]\{x(t)\}=\{f(t)\right\}$$

(2) $$s^{2}[M]+s[C]+[K]\{X(s)\}=\{f(s)\}$$

where [M], [C] and [K] respectively represent the mass, damping and stiffness matrix.

**Figure 6:**
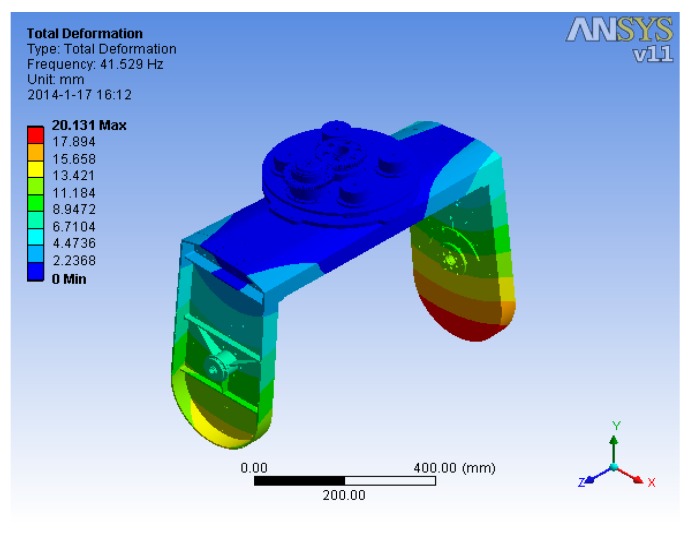
The first order mode of ISP structure.

The solution of these equations leads to an eigenvalue problem that can be solved in terms of the modal parameters. Eventually, the natural frequency and shape of each order for a system with N Degrees of Freedom (DOFs) can be obtained. Figure 6 and Table 1 shows the FEA dynamic results that satisfy the dynamic design requirements.

**Table 1:** The FEA results of the prior six modes at natural frequency.

Models	1	2	3	4	5	6
Frequency/Hz	41.5	45.2	55.1	74.8	121.8	185.2

## 4. System Dynamics Modeling and Analysis

### 4.1. System Dynamics Modeling

**Figure 7:**
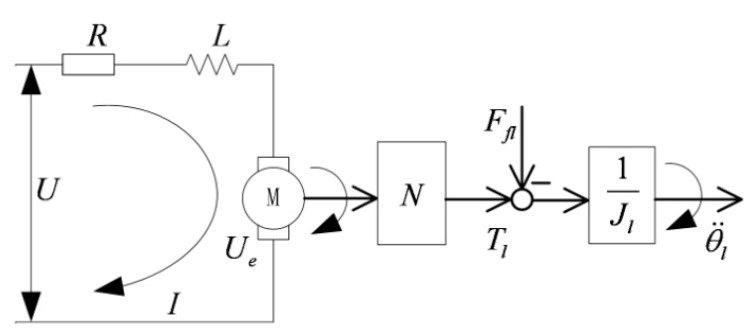
Block diagram for a simplified gear drive system with fixed transmission ratio.

Since the maximum required velocity of an ISP is usually low, gearing are used to reduce the size and weight of the actuator, particularly when the torque requirements are demanding [23]. Therefore, to meet the requirements of highly driving torque, heavy load and small size, *etc.*, both gimbals are designed to be rotated by indirect drive motors linked to each gimbal through gear-trains. The gear drive system model, as shown in Figure 4, can be simplified with a fixed transmission ratio, as shown in Figure 7.

According to the current balance equation and the torque balance equation, at the side of the motor output axis, the following equations are satisfied:

(3) $$U=IR+L\frac{dI}{dt}+k_{b}\omega_{m}$$

(4) $$J_{m}{\ddot{\theta }}_{m}+b_{m}\omega_{m}=T_{m}-\tau_{l}-F_{fm}$$

(5) $$T_{m}=k_{T}I$$

where *U*, *I*, *R* and *L* are respectively the armature voltage, current, resistance and inductance of torque motor; *k_b_* is the back EMF constant of motor, $\omega_{m}$ is angular velocity of motor; *J_m_* is the rotational inertia of motor, $\theta_{m}$ is angular displacement of motor, *b_m_* is equivalent damping of motor, *T_m_* is electromagnetic torque of motor, *τ_l_* is load torque at the side of motor output axis, *F_fm_* is the inner friction torque of motor; *k_T_* is the torque coefficient of motor.

### 4.2. Gimbal Dynamics

According to references [20,21], the traditional Newton-Euler method is used to model the two-axis ISP in this paper. The basic Newton-Euler equation for a rotational rigid body is described by

(6) $$\sum M=J\dot{\omega }+\omega \times J\omega$$

where ∑M is the resultant moment of force applied to a rigid body, J is the inertia moment. ω and $\dot{\omega }$ are the absolute angular rate and angular acceleration, respectively. Assuming that the two gimbals are symmetrical, then the dynamic models of the two gimbals can be described as below.

#### 4.2.1. Dynamic Model of the Pitch Gimbal

Based on the coordinate definition shown in Figure 4, we assume that the resultant moment of force applied to the pitch gimbal is ∑M_E_, dynamic model of the pitch gimbal is given by:

(7) $$\sum M_{E}=J_{E}{\dot{\omega }}_{IE}^{E}+{\dot{\omega }}_{IE}^{E}\times J_{E}{\dot{\omega }}_{IE}^{E}$$

Because the pitch gimbal only has rotational freedom in the direction of x_E_, its dynamic model around axis x_E_ is given by:

(8) $$M_{mEx}-M_{dEx}=J_{Ex}{\dot{\omega }}_{IEx}^{E}+(J_{Ez}-J_{Ey})\omega_{IEy}^{E}\omega_{IEz}^{E}$$

#### 4.2.2. Dynamic Model of the Azimuth Gimbal

Assume that the resultant moment of force applied to the pitch gimbal is ∑M_A_, dynamic model of the azimuth gimbal is given by:

(9) $$\sum M_{A}=J_{A}{\dot{\omega }}_{IA}^{A}+\omega_{IA}^{A}\times J_{A}\omega_{IA}^{A}$$

Then dynamic model of the azimuth gimbal around axis z_A_ is given by:

(10) $$M_{mAz}-M_{dAz}+M_{E/Az}=J_{Az}{\dot{\omega }}_{IAz}^{A}+(J_{Ay}-J_{Ax})\omega_{IAx}^{A}\omega_{IAy}^{A}$$

where M_E/Az_ refers to the coupling torque between the pitch gimbal and the azimuth gimbal expressed in the direction of z_A_, and it is given by:

(11) $$M_{E/Az}=\sin\theta_{e}[J_{Ey}{\dot{\omega }}_{IEy}^{E}+(J_{Ex}-J_{Ez})\omega_{IEx}^{E}\omega_{IEz}^{E}]+\cos\theta_{e}[J_{Ez}{\dot{\omega }}_{IEz}^{E}+(J_{Ey}-J_{Ex})\omega_{IEx}^{E}\omega_{IEy}^{E}]$$

Thus, dynamic model of the two-axis ISP can be described as:

(12) $$\left\{ \begin{array}{l} {\dot{\omega }}_{IEx}^{E}=\frac{M_{mEx}-M_{dEx}-(J_{Ez}-J_{Ey})\omega_{IEy}^{E}\omega_{IEz}^{E}}{J_{Ex}}\\ {\dot{\omega }}_{IAz}^{A}=\frac{\left\{\begin{array}{l} M_{mAz}-M_{dAz}-(J_{Ay}-J_{Ax})\omega_{IAx}^{A}\omega_{IAy}^{A}-\sin\theta_{e}[J_{Ey}{\dot{\omega }}_{IEy}^{E}+(J_{Ex}-J_{Ez})\omega_{IEx}^{E}\omega_{IEz}^{E}]\\ +\cos\theta_{e}[J_{Ez}{\dot{\omega }}_{IEz}^{E}+(J_{Ey}-J_{Ex})\omega_{IEx}^{E}\omega_{IEy}^{E}] \end{array}\right\}}{J_{Az}} \end{array}\right.$$

## 5. Co-Modeling of the Mechatronic System

### 5.1. The Principle of Electromechanical Co-Simulation

**Figure 8:**
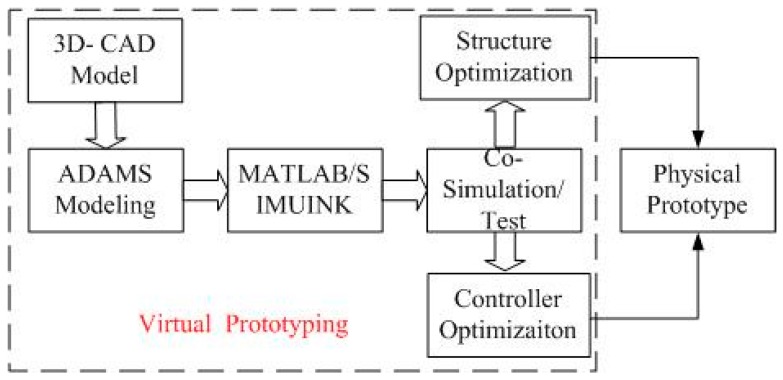
Flow chart of electromechanical co-simulation.

The co-modeling of the mechatronic system involves two aspects: mechanical system modeling and control system modeling. When the mechanical models are established, the dynamic performance of the system can be improved by mode simulation analysis and structural optimization, which is the basis of the control system design. When the control system parameters are optimized, the high-fidelity VP based on ADAMS™ and SIMULINK^®^ cooperation can be used for the performance testing, visualization simulation of the electromechanical system, control system parameter optimization, and reliability prediction, *etc.* Eventually, high-fidelity parameters for the control system can be obtained so that a control system with high accuracy and stabilization is achieved. In conclusion, the ADAMS™ model can be used for a control design that is developed in the SIMULINK^®^ environment. State variables of the ADAMS™ model are connected with the control system in SIMULINK^®^ and the whole VP is tested [12]. Figure 8 shows the flow chart of electromechanical co-simulation.

### 5.2. Mechanical System Modeling

#### 5.2.1. ADAMS™ Environment Analysis

The co-simulation of the mechatronic system is an important part of VP technology. In the traditional modeling and simulation, the mechanical system is generally assumed to be an ideal model, in which many factors are not taken into good account, such as the installation error, mass unbalance, and so on. In particular, the coupling between the base angular motion and the mass center bias cannot be included in the modeling.

The ADAMS™ environment is able to provide a multi-body model for static, kinematic and dynamic analysis [12]. Moreover, ADAMS™ provides a way to analyze complex electromechanical systems by co-simulation. ADAMS™/Controls is a plug-in for ADAMS™ that helps one add sophisticated controls to the ADAMS™ model.

#### 5.2.2. ADAMS™-Based Mechanical System Modeling

**Figure 9:**
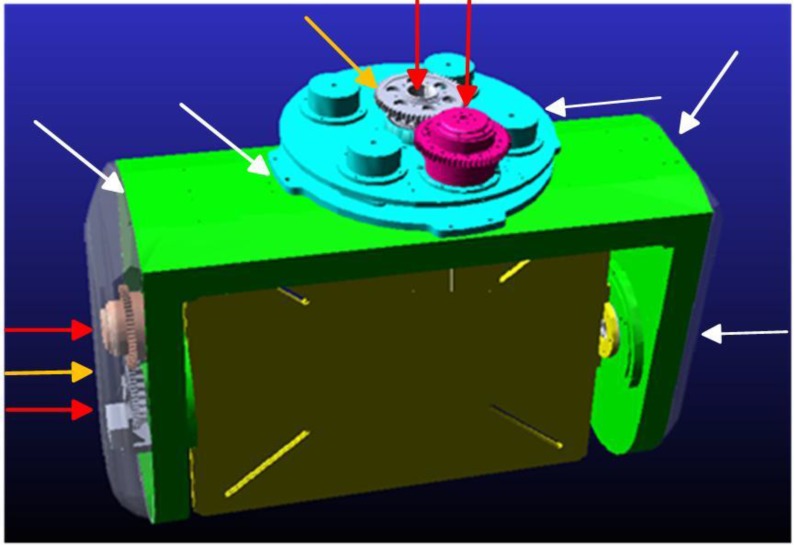
Constraints addition of ISP under ADAMS.

**Figure 10:**
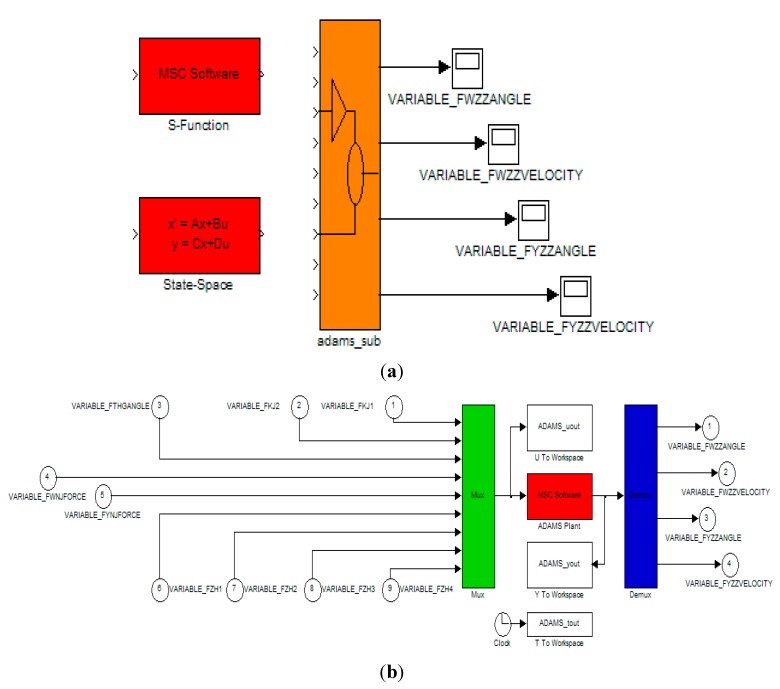
Co-simulation interface module of the ISP: (**a**) connection between ADAMS™ and SIMULINK^®^ models; and (**b**) ADAMS™ module in co-simulation model.

First, a three-dimensional structural model of the ISP established in SOLIDWORKS^®^ is input into the ADAMS™ environment. Then, an ADAMS™-based mechanical system model can be obtained, whose procedures are as follows:

(1) Setting Parameters. Add the necessary parameters into the ADAMS™ model, such as the gravitational acceleration, material properties, *etc.* Furthermore, adjust the center of mass to minimize the mass unbalance, which is helpful to reduce the structural coupling effects.

(2) Adding constraints, driving moments and loading. The different movement constraints need to be defined to reflect the system movement. Through constraints, the components are associated with each other and the relative movement is limited [17]. The ISP contains five fixed pairs and five transmission pairs. Two gear pairs are respectively set for azimuth and pitch transmission links to realize the connection from the rotation of the DC motor to the movement of two gimbals. Figure 9 shows the constraints addition of ISP under the ADAMS™ environment.

(3) Defining state variables. In order to exchange information between the mechanical and control systems, we need to define the state variables and build up the interfaces between input and output. State variables are the key links of the internal information inflow and outflow in the ADAMS™ model. State variables for the system output are the angular velocities and rotational angles of the azimuth gimbal and pitch gimbal.

(4) Establishing interfaces. The principle of the co-simulation between ADAMS™ and SIMULINK^®^-Driveline is that digital signals which are produced in one step in ADAMS™ and SIMULINK^®^ are respectively transferred through interfaces. This process continues until the simulation ends [17].

(5) Obtaining the co-simulation interface module. The ADAMS™ model is fed under the SIMULINK^®^ environment to be analyzed. The model is used in the control scheme to predict the behavior of the system using a PID controller [27]. Figure 10 shows the co-simulation interface of the ISP.

### 5.3. Control System Modeling

#### 5.3.1. SIMULINK^®^ Environment Analysis

In order to execute a co-simulation between ADAMS™ and MATLAB^®^, definition of an acceptable format for the inputs and outputs of each program is required. The objective of co-simulation is to make a connection so that any change in one of the programs affects the other one [28]. The response speed and stabilization performance of an ISP can be influenced seriously by the mechanical system. MATLAB^®^ is used in the model construction of the control system, and the mechanical model of ADAMS™ is connected into the MATLAB^®^ control model. Thus, co-modeling between the mechanical dynamics and the control system is performed. Based on electromechanical co-modeling, the ISP control parameters can be optimized by co-simulation of the mechatronic system.

#### 5.3.2. SIMULINK^®^-Based Control System Modeling

(1) Control system model

The transfer function analysis in control system is as follows: the drive motor can be equivalent to LR circuit, which is composed of inductors and resistors in series. The transfer function is:

(13) $$G_{m}=\frac{K_{m}}{T_{e}s+1}$$

where $T_{e}$ is the electromagnetic time constant, $T_{e}=\frac{L}{R}$; $K_{m}$ is the motor current coefficient, $K_{m}=\frac{1}{R}$.

The equivalent transfer function of motor BEMF is $wk_{e}$, where w is the motor speed, $k_{e}$ is the BEMF coefficient of the motor.

The PWM module of a drive motor can be equivalent to a first-order inertia link:

(14) $$G_{pwm}=\frac{U}{T_{pwm}s+1}$$

where U represents the supply voltage of PWM module, and $T_{pwm}$ is the period of carrier wave of PWM output. The current measurement module is taken as the proportion of links $K_{f}$, in which the Hall sensor is employed.

**Figure 11:**
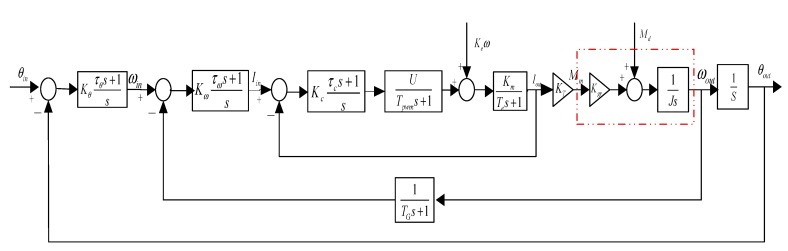
Block diagram of three closed-loop compound PID control scheme of two-axis ISP.

Rate gyro can be taken as a first-order inertia link, $\frac{1}{T_{G}s+1}$, where $T_{G}$ is the sampling period. Based on the analysis above, we can obtain the three closed-loop compound PID control scheme of two-axis ISP, as shown in Figure 11. In Figure 11, the mechanical part is outlined with a red dashed line.

(2) SIMULINK^®^-based modeling

From Figure 11, we can first analyze the mechanical parts of the three closed-loop compound PID control scheme of the two-axis ISP: for the “Adams-sub” module established in ADAMS™, its input part is the motor torque, and its output part is the angular position and speed of gimbals resolved by the module automatically in real-time. For the system model established by MATLAB^®^, its input part is the output part of “Adams-sub”, *i.e.*, the angular position and speed of gimbals resolved by the module automatically in real-time under ADAMS™.

**Figure 12:**
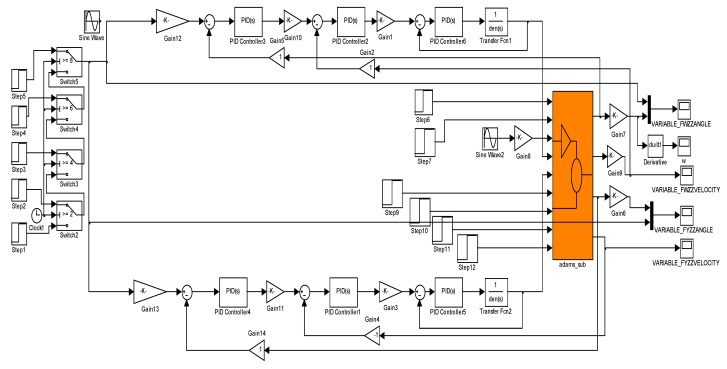
ISP model in SIMULINK^®^.

Figure 12 is the ISP model produced in SIMULINK^®^. By inputting the “Adams-sub” block in the SIMULINK^®^ environment, the ISP model is suitable for control and motion simulation as a defined system in MATLAB^®^. When the co-simulation model is established, it is necessary to simplify the transfer function models of the electrical part. For example, the function of motor BEMF, the time delay of the power-drive, and the time delay of the gyro signal can be ignored since their effects are too weak to be considered.

## 6. Co-Simulation and Testing

### 6.1. Dynamic Performance and Steady-State Accuracy Testing

#### 6.1.1. Step Response

**Figure 13:**
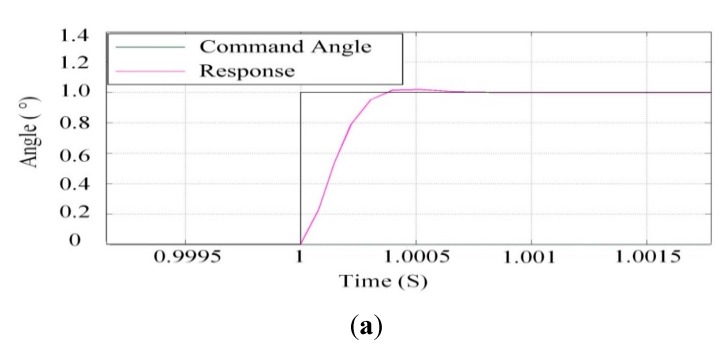

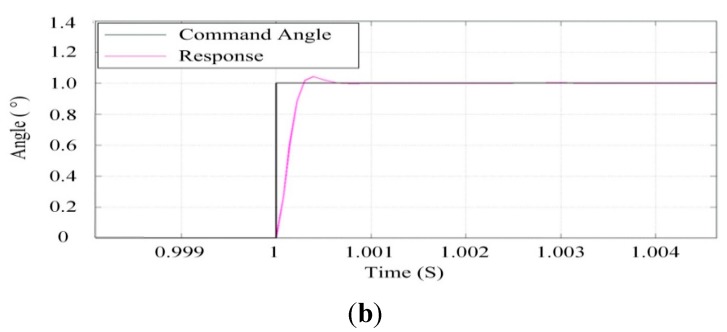
Step response of co-simulation: (**a**) the azimuth gimbal; (**b**) the pitch gimbal.

Figure 13 shows the step response of the azimuth gimbal and the pitch gimbal under co-simulation based on ADAMS™ and SIMULINK^®^ cooperation.

The black curve represents the set angular position and the purple curve is the response of the ISP. The interaction time interval is 0.0001 s. It can be seen that with help of the control parameters of the electromechanical simulation model, the ISP can realize high response speed and provide steady-state accuracy with mechatronic system co-simulation.

#### 6.1.2. Sinusoidal Response

**Figure 14:**
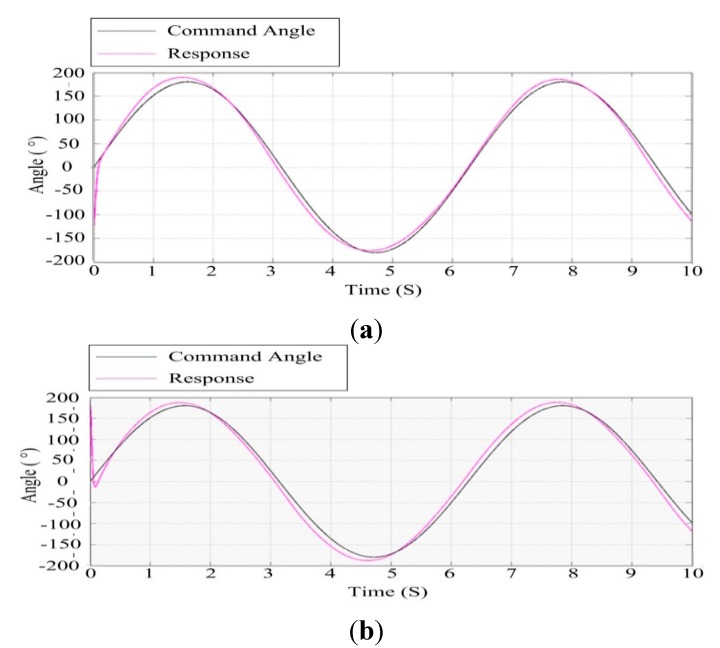
The response curve to a sinusoidal angular command in the electromechanical co-simulation model: (**a**) the azimuth gimbal; (**b**) the pitch gimbal.

Figure 14 shows the response curve of the azimuth and pitch gimbals for the sinusoidal angular input command under co-simulation based on ADAMS™ and SIMULINK^®^ cooperation. Likewise, the black curve represents the set angular position and the purple curve is the response of the ISP. It can be seen that with the help of the control parameters of the electromechanical simulation model, the ISP can track the command angle rapidly and accurately, by which the high stabilization performance can be attained.

#### 6.1.3. Simulation Testing under Movable Base Conditions

**Figure 15:**
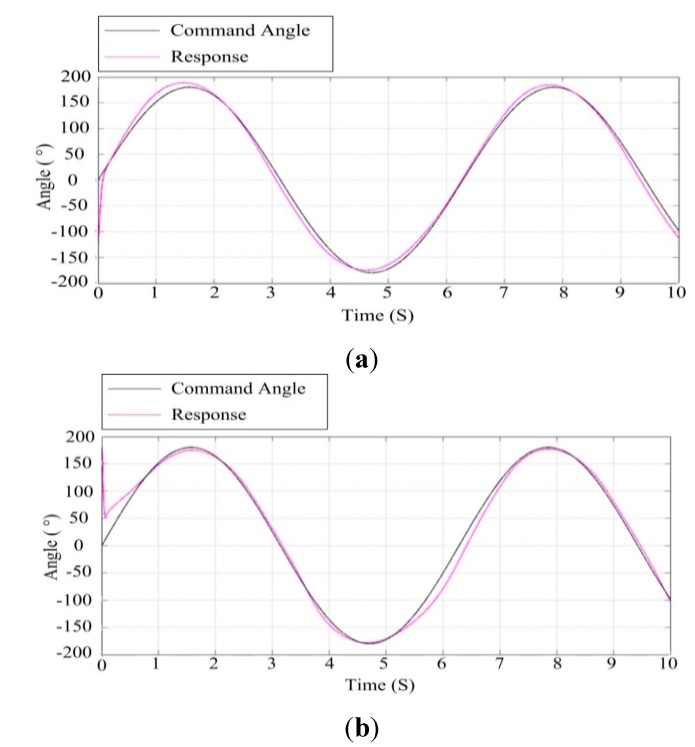
The response curve under roll swing of 10° sinusoidal angle of the UA: (**a**) the azimuth gimbal; (**b**) the pitch gimbal.

Due to fact the pitch axis of the ISP coincides with the roll axis of the UA, the roll swing of the UA corresponds to the input of the ISP pitch disturbance. Therefore, it is necessary to test the control performance of the ISP under movable base conditions by simulating the roll swing of the UA at various angles. Figure 15 shows the response curve for a 10° sinusoidal angle roll swing of the UA under co-simulation. It can be seen that under this movable base condition, the ISP has good tracking accuracy whether for azimuth gimbal or pitch gimbal.

**Figure 16:**
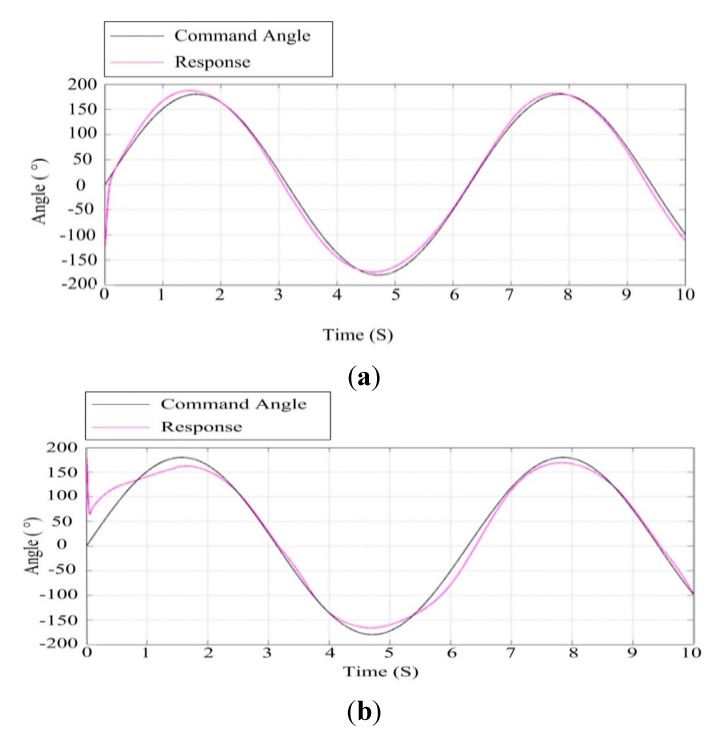
The response curve under 30° sinusoidal angle roll swing of the UA: (**a**) the azimuth gimbal; (**b**) the pitch gimbal.

Figure 16 shows the response curve for a 30° sinusoidal angle roll swing of the UA under co-simulation. It can be seen that under this movable base condition, the azimuth gimbal still has good tracking accuracy, but the tracking accuracy of the pitch gimbal begins to decrease since there is an obvious deviation between the command and its response. This means that when the roll swing of the UA is up to about 30°, the ISP no longer has enough capability to isolate the UA disturbance with high accuracy.

### 6.2. Analysis of the Effect of Wind Speed

The wind force is an important factor influencing the performance of an ISP. Therefore, performance testing of the ISP under the wind force is simulated. According to the Bernoulli wind-pressure equation, we get:

(15) $$F=\frac{v^{2}}{1.6}S$$

where F is the wind force on the object, v is the wind speed, S is swept area of the object. The total swept area of the ISP is approximately 0.5 m^2^ and the wind speed is 10 m/s, so we get F is about 31 N.

**Figure 17:**
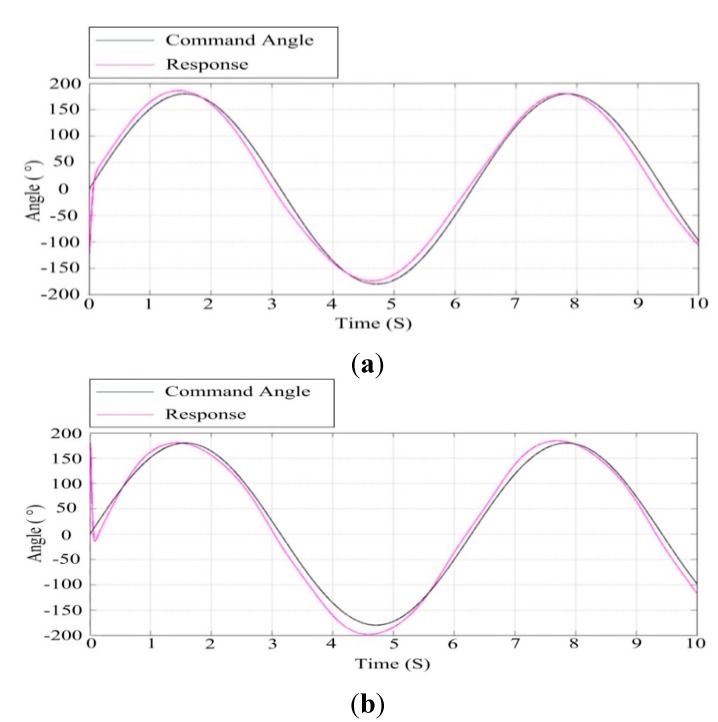
Response curves of ISP at the wind speed of 10 m/s: (**a**) the azimuth gimbal; (**b**) the pitch gimbal.

**Figure 18:**
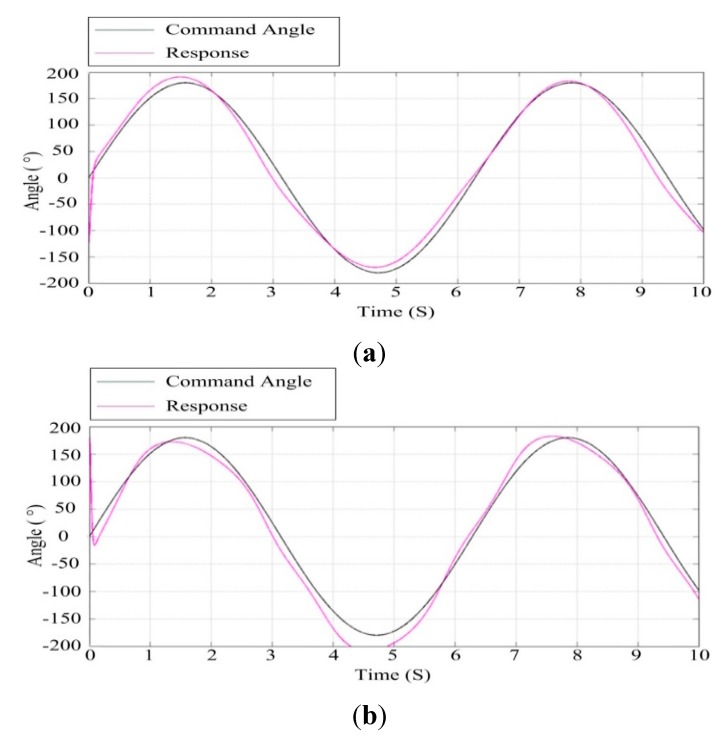
Response curves of the ISP at the speed of 14 m/s: (**a**) the azimuth gimbal; (**b**) the pitch gimbal.

Figure 17 and Figure 18 show the results of simulation tests under different wind speeds by applying a wind force disturbance to the ISP. Comparing Figure 17 with Figure 18, we can see that when the wind speed is 10 m/s, the azimuth gimbal and the pitch gimbal of the ISP have better tracking performance than that when the wind speed is 14 m/s. This means when the wind speed is larger, the tracking performance of the ISP will decrease, particularly for the pitch gimbal. Therefore, we can determine the wind speed reliability range to guarantee the stabilization performance of ISP.

## 7. Experimental Verification

### 7.1. Testing Under Movable Vehicle Conditions

**Figure 19:**
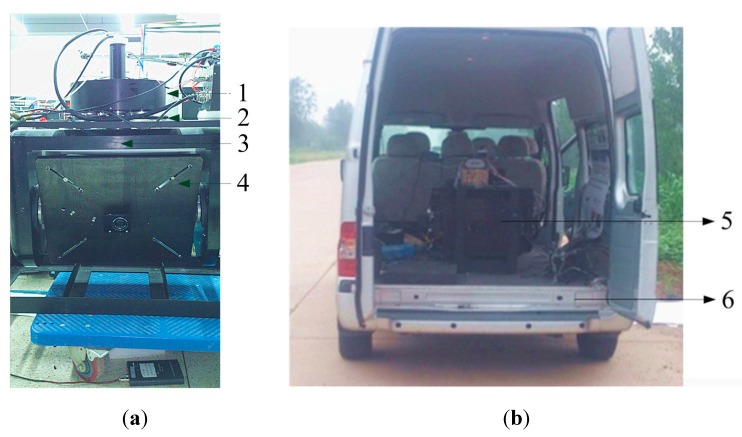
Picture of the ISP during experiments under movable base conditions: (**a**) ISP and (**b**) movable vehicle. 1-Loads; 2-Base; 3-Azimuth gimbal; 4-Pitch gimbal; 5-ISP; 6-Movable vehicle.

Figure 19 shows a picture of our real experimental two-axis ISP system and the movable vehicle. The ISP originally works with the control parameters optimized by co-simulation. Then, the parameters are further optimized and improved based on actual electromechanical tests. The eventual control parameters are based on those of co-simulation and only differ very little from the simulation results. This illustrates that the co-simulation is an important way to design control systems with high performance.

**Figure 20:**
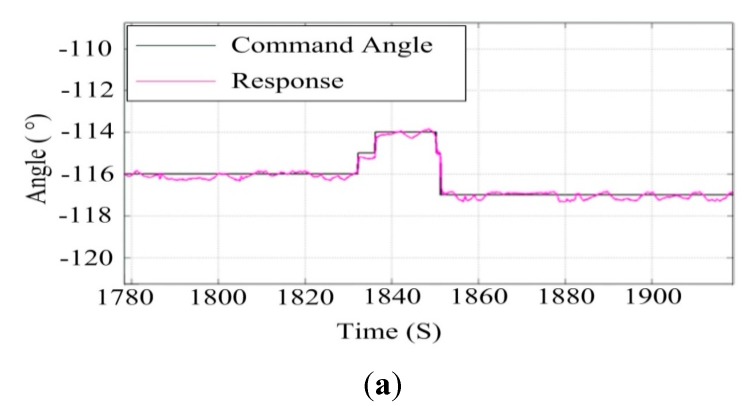

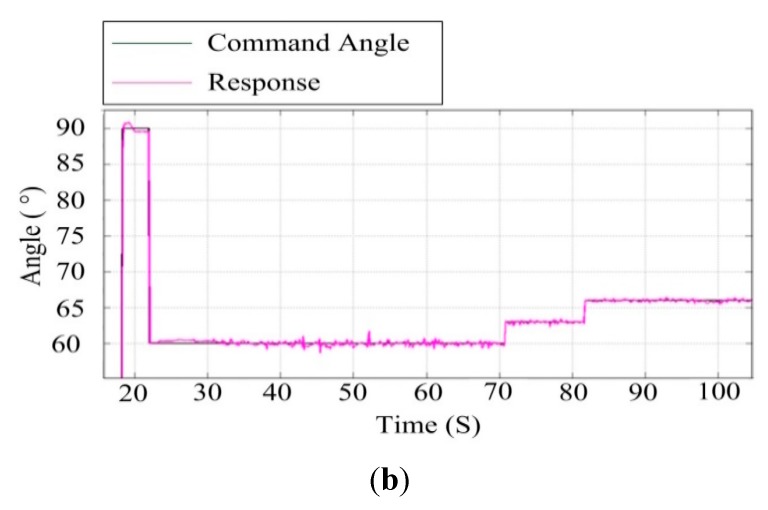
The tracking accuracy of the ISP in a real movable vehicle experiment: (**a**) the azimuth gimbal; (**b**) the pitch gimbal.

Figure 20 shows the tracking results of the ISP in a real movable vehicle experiment. It can be seen that the ISP has high tracking accuracy to the instruction command that makes it track stably to the target. The steady pointing accuracy is less than 0.5° (RMS) under the conditions where the tracking angular speed is less than 40°/s.

### 7.2. Testing the UA in Air

In ISP design, the wind force interaction is an important disturbance. If an ISP and payload are exposed to the vehicle’s aerodynamic wind stream, direct buffeting occurs [29]. However, the torque due to wind is modeled using a moment coefficient which is quite difficult to estimate, and the model of the air movement in the flight environment is so complex that many theories have been formed, such as the Von Karmen vortices. Therefore, to simplify the design, the operating disturbances including wind force are generally modeled as equivalent torque disturbances [29]. In this paper, thus, we use a simple wind force model to simulate the ISP performance when the airship is subjected to a certain wind speed. The air experiments were carried out under good weather conditions with only a little wind speed.

**Figure 21:**
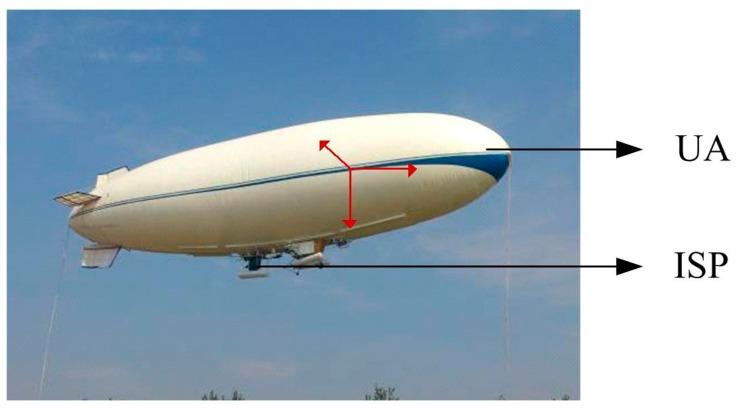
The experiments with a two-axis ISP system on a UA in the air.

**Figure 22:**
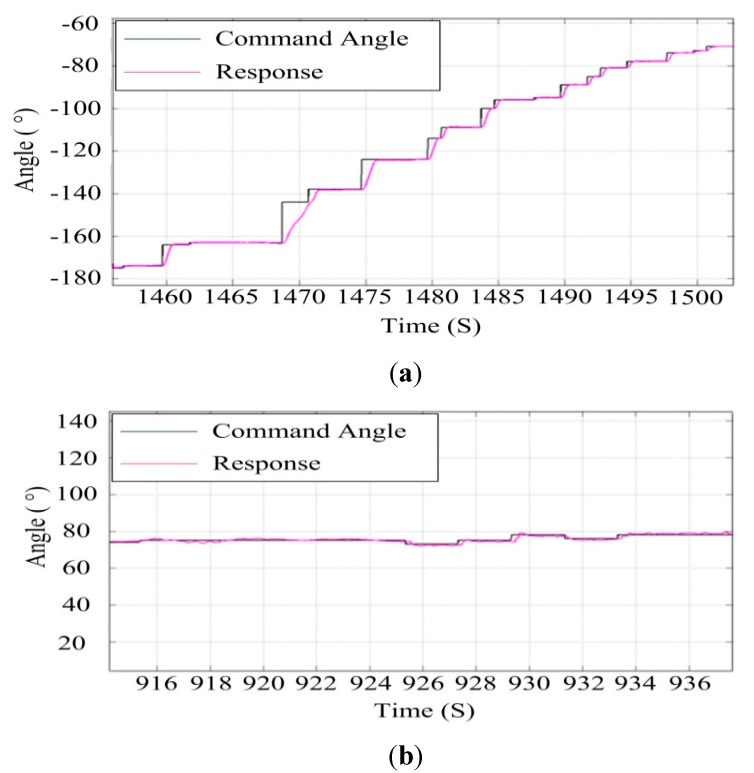
The tracking results of the ISP in the experiments on a real UA in the air: (**a**) the azimuth gimbal; (**b**) the pitch gimbal.

Figure 21 shows a picture of an experiment with a two-axis ISP system on an UA in air. The control parameters are those determined in the movable vehicle experiments. Figure 22 shows the ISP tracking results of the experiments on a real UA in the air. It can be seen that under the real conditions of the UA in the air, the ISP still has high tracking accuracy with the instruction command, which is similar to the results in the movable vehicle experiments. The steady-state accuracy of the system under UA in air is 0.6° (RMS), and the velocity under the tracking angular is 20 °/s.

## 8. Conclusions

In this paper, to realize high control performance of a two-axis ISP applied in UAs, a method based on co-simulation of the mechatronic system is proposed to optimize the control parameters. Depending on the built three-dimensional CAD structural model, the dynamics model is established under an ADAMS™ environment, by which the main dynamic parameters are obtained through simulation analysis. Then, the dynamic parameters are input into the SIMULINK^®^ controller to simulate and test the performance of the control system to optimize the control parameters. Experiments were carried out to verify the method. The results show that the proposed method is effective in that it can obviously improve the accuracy and reliability of the ISP.
